# Knowledge and Awareness of Pediatric Obstructive Sleep Apnea Among the Adult Population in Al-Ahsa, Saudi Arabia: A Cross-Sectional Study

**DOI:** 10.7759/cureus.112106

**Published:** 2026-07-05

**Authors:** Anwar Alsultan, Mohammed Alanazi, Thekra M Alqarafa, Mousa M Aljassim, Abdullah Aljamah

**Affiliations:** 1 Family Medicine, Primary Health Care-Ministry of Health (PHC-MOH), Al-Ahsa, SAU; 2 Family Medicine, Alahsa Health Cluster, Al-Ahsa, SAU; 3 Family Medicine, Alahsa Family Medicine Academy, Ministry of Health Holdings, Al-Ahsa, SAU

**Keywords:** awareness, knowledge, obstructive sleep apnea, pediatric, saudi arabia

## Abstract

Background

Pediatric obstructive sleep apnea (OSA) causes severe neurobehavioral and developmental complications, yet public awareness remains understudied in regional Saudi Arabia. This study evaluated knowledge of pediatric OSA among the general population in Al-Ahsa, Saudi Arabia, and identified its sociodemographic determinants.

Methodology

This cross-sectional study surveyed the adult population of Al-Ahsa between July 30, 2025, and May 30, 2026. Utilizing a convenience sampling method, data were gathered from 393 participants via a pre-tested, validated, self-administered questionnaire. Knowledge levels were dichotomized into good (greater than 70%) or poor (70% or less). Statistical analyses, encompassing both descriptive and inferential statistics, were performed using IBM SPSS Statistics for Windows, Version 27 (Released 2019; IBM Corp., Armonk, New York, United States).

Results

Among 393 participants, 197 (50.1%) were male, 278 (70.7%) were university-educated, and most of them (378; 96.2%) demonstrated poor overall knowledge of pediatric OSA. While 308 (78.4%) knew it was treatable, 235 (59.8%) were unaware of its impact on academic performance, and only 23 (5.9%) recognized its definitive respiratory criteria. Higher knowledge scores significantly correlated with advanced education (p < 0.001), being married (p = 0.022), and fewer children (p = 0.001). Social media was the main information source (32.1%), while 42.7% had never heard of the condition.

Conclusion

Public knowledge and awareness of pediatric OSA in Al-Ahsa is critically deficient. Target campaigns utilizing digital media and clinical specialists are urgently needed.

## Introduction

Obstructive sleep apnea (OSA) is a condition marked by recurrent instances of either partial or total blockage of the airway while sleeping [1]. The severity of the condition is influenced by how often these episodes occur [2].

Pediatric OSA usually presents with consistent snoring, along with cognitive and behavioral issues like learning challenges, attention deficits, hyperactivity, and impulsiveness, which may be associated with a diagnosis of attention deficit hyperactivity disorder [3]. The occurrence rate among children is between 2% and 4% [4]. The majority of children fall within the age range of two to eight years, which is linked to the proportionate size of the lymphatic tissue in the upper airways [5]. It has been established that craniofacial abnormalities and adenotonsillar hypertrophy are factors that raise the occurrence of OSA [6]. Like adults, obese children seem to have a higher risk for developing sleep-disordered breathing, and the severity of OSA appears to correlate with the level of obesity [7]. With a more complex spectrum of symptoms, there is no obvious relationship between the severity of the clinical presentation and daytime symptoms in children with OSA [2]. Untreated OSA is linked to a range of complications such as neurobehavioral problems, delays in growth and development, cardiovascular dysfunction, and resistance to insulin [8]. The diagnosis of pediatric OSAS requires a thorough clinical history, focusing on physical examinations, especially in the ENT region, as well as nocturnal and daytime symptoms and comorbidities, along with specific questionnaires given to parents. Nocturnal oximetry and portable polysomnography are essential components of the evaluation [5].

Numerous studies indicate a relationship between treating pediatric OSA and enhancements in cognition, behavior, quality of life, and sleep [3]. In a study carried out in Saudi Arabia, a total of 1,000 participants showed that the overall knowledge level was poor for 80.7% of the sample and good for 19.3% [9]. In a study conducted in Jeddah, it was revealed that most parents visiting the pediatric clinic in Jeddah possessed little knowledge regarding the symptoms and risk factors associated with pediatric OSA [3]. In another study conducted in Jazan, researchers found considerable gaps in awareness of pediatric OSA symptoms and their effects on children's academic success and mental well-being. Although knowledge was limited, parents showed a willingness to engage in early intervention for pediatric OSA. The research indicates that parents with a larger number of children demonstrated greater understanding of POSA [10]. Another research study revealed that the understanding of pediatric OSA among graduating medical and dental students in Saudi Arabia, as well as recent graduates, was not satisfactory [2].

The findings highlight the essential need to raise awareness and improve education regarding this condition among both the public and healthcare professionals in Saudi Arabia. There are limited studies published that focus on awareness of pediatric OSA in Saudi Arabia, and no prior research has evaluated the level of awareness and understanding of pediatric OSA in Al-Ahsa. The primary aim of this study is to assess the knowledge and awareness of pediatric OSA among the general population in Al-Ahsa and identify potential factors associated with their knowledge level.

## Materials and methods

Study design and population

This cross-sectional study was conducted among the adult population of Al-Ahsa, Saudi Arabia, from July 30, 2025, to May 30, 2026. The inclusion criteria required participants to be Saudi or non-Saudi residents of Al-Ahsa, of either gender, and aged 18 years or older. Based on a 95% confidence level, a 5% margin of error, and a 50% response distribution, the minimum required sample size was determined to be 385 participants using the Raosoft online calculator. To ensure adequate statistical power and account for potential data loss from incomplete responses, the final sample size was expanded to encompass 393 participants.

Study tool

Data were gathered using a pretested, validated online self-administered questionnaire distributed across various social media platforms after permission from a previously published study [3]. The instrument demonstrated strong internal consistency, with a Cronbach’s alpha of .82. Furthermore, a pilot study yielded good internal consistency, with a Cronbach’s alpha of .73. The structured questionnaire was divided into two primary sections. The first section gathered data on the sociodemographic characteristics of the participants. The second section evaluated knowledge and awareness regarding pediatric OSA, specifically focusing on its definition, clinical symptoms, associated risk factors, and general information. Assessment scoring was based exclusively on the 30 items within this second section. Participant knowledge levels were then categorized based on their total scores: individuals scoring greater than 70% were allocated to the good knowledge cohort, while those scoring 70% or less were classified into the poor knowledge cohort.

Statistical analysis

Data were collected via Google Forms, coded into Microsoft Excel, and subsequently transferred to IBM SPSS Statistics for Windows, Version 27 (Released 2019; IBM Corp., Armonk, New York, United States) for statistical analysis. Both descriptive and inferential statistics were performed. Categorical variables were expressed as frequencies and percentages, while continuous variables including the total knowledge score were presented as means and standard deviations (SD). Data were visualized using tables and figures. For inferential analysis, an independent-samples t-test was conducted to compare mean knowledge scores across dichotomous variables. One-way analysis of variance (ANOVA) was utilized to evaluate differences in mean knowledge scores across categorical variables with more than two groups. Additionally, a multiple linear regression analysis was performed to identify independent predictors of knowledge. The normality of the data distribution was assessed prior to parametric testing. For all statistical tests, a p-value ≤ 0.05 was considered statistically significant.

Ethical consideration

Ethical approval for this study was formally granted by the IRB of Alahsa Health Cluster on 15-07-2025 under IRB Log No.: 1907-EP-2025. Prior to participation, informed consent was obtained from all respondents, who were explicitly notified that participation was entirely voluntary and that they maintained the right to withdraw from the study at any stage without penalty. To ensure strict confidentiality, all collected data were anonymized, and no personally identifiable information will be disclosed or published in the final results.

## Results

Table 1 shows that this study included 393 participants, with 197 (50.1%) male participants, more than half of them aged between 18 and 40 years, and a high level of educational attainment (278 (70.7%) with a bachelor’s degree or higher). Statistical analysis revealed that while age and gender did not influence awareness, educational level (p < .001), number of children (p = .001), and marital status (p = .022) were significant. Participants with postgraduate education followed by graduate education demonstrated the highest mean knowledge scores. Participants with eight or more children had a lower knowledge score. In addition, married participants scored higher than single, divorced, or widowed participants.

**Table 1:** Sociodemographic characteristics and mean knowledge score by sociodemographic variables N: Frequency, %: Percentage, SD: standard deviation, OSA: obstructive sleep apnea, and *: Significant ^a^: one-way ANOVA test, ^b^: independent t-test, F / t value: for one-way ANOVA test and independent t-test, respectively

Variables	Categories	N (%)	Knowledge Score (Mean ± SD)	F /t value	p-value
Age Group ^a^	18 – 30	108 (27.5%)	11.32±5.22	1.97	0.098
	31 – 40	110 (28.0%)	11.57±4.73		
	41 – 50	92 (23.4%)	10.84±4.33		
	51 – 60	61 (15.5%)	11.02±4.24		
	More than 60	22 (5.6%)	8.64±3.09		
Sex ^b^	Male	197 (50.1%)	10.95±4.66	.305	0.581
	Female	196 (49.9%)	11.21±4.67		
Marital Status ^a^	Single	70 (17.8%)	10.52±5.18	3.25	0.022*
	Married	266 (67.7%)	11.54±4.51		
	Divorced	50 (12.7%)	9.81±4.33		
	Widower	7 (1.8%)	8.43±4.94		
Education Level ^a^	Uneducated	6 (1.5%)	8.58±4.07	6.25	< .001*
	High school	109 (27.7%)	9.96±4.11		
	Bachelor's degree	239 (60.8%)	11.28±4.51		
	Postgraduate studies	39 (9.9%)	13.38±6.04		
Number of Children ^a^	None	89 (22.6%)	11.19±5.10	4.02	0.001*
	1 – 3	139 (35.4%)	11.37±4.87		
	4 – 7	157 (39.9%)	11.05±4.10		
	8 or more	8 (2.0%)	5.56±3.39		
Do you have child suffering from OSA ^b^	Yes	337 (85.8%)	10.92±4.76	-1.67	0.096
	No	56 (14.2)	12.04±3.91		

Table 2 details the participants' responses to specific knowledge items regarding childhood OSA. More than three-quarters of them (308; 78.4%) acknowledged that childhood OSA is treatable, 317 (80.7%) recognized that early intervention and management can mitigate the risk of complications, and 305 (77.6%) agreed that participants' awareness is instrumental in reducing the burden on families and the general population. More than half of the participants were unaware that OSA can impact school performance (235; 59.8%) or influence attention and behavior (215; 54.7%). Furthermore, a substantial majority, 272 (69.2%), did not know that children with OSA have a higher prevalence of depression, and 206 (52.4%) were unaware of the role genetic factors play in the etiology of the disorder. Only 23 (5.9%) can truly answer the definition of OSA. Regarding community awareness preferences, participants identified a multifaceted approach to health education. Less than three-quarters (288; 73.3%) prioritized consulting a specialist doctor. This was followed closely by voluntary awareness campaigns (275; 70.0%) and more than half of them (215; 54.7%), recognized the internet and social media as vital tools for dissemination.

**Table 2:** Knowledge items and community awareness preferences concerning OSA OSA: Obstructive sleep apnea

Knowledge items		Frequency	Percentage
Do you know that childhood obstructive sleep apnea can affect a child’s school performance?	No	235	59.8
	Yes	158	40.2
Do you know that children with obstructive sleep apnea have a higher prevalence of depression than other children?	No	272	69.2
	Yes	121	30.8
Do you know that childhood obstructive sleep apnea affects attention and behaviour?	No	215	54.7
	Yes	178	45.3
Do you think that the genetic factor can have a role in the cause of childhood obstructive sleep apnea?	No	206	52.4
	Yes	187	47.6
Do you think that childhood obstructive sleep apnea can be treated?	No	85	21.6
	Yes	308	78.4
Do you think early intervention and management can reduce the possible risk of complications in children with obstructive sleep apnea?	No	76	19.3
	Yes	317	80.7
Do you think parents' awareness about childhood obstructive sleep apnea can help reduce the burden on them and the population?	No	88	22.4
	Yes	305	77.6
Awareness that OSA is characterized by episodes of recurrent breathing pauses (apnea) during sleep	No	370	94.1
	Yes	23	5.9
In your opinion, what is the most appropriate way to raise community awareness about obstructive sleep apnea in children?			
Consulting a specialist doctor		288	73.3
The Internet and social media		215	54.7
Voluntary awareness campaigns		275	70.0

Table 3 showing the regression analysis reveals that education level (β= .200, p < .0001) and prior knowledge of a child with OSA (β = 1.422, p = .033) are the only significant positive predictors of increased pediatric OSA knowledge. Demographic factors including age, sex, marital status, and having children were not found to have a statistically significant association with knowledge levels in this model.

**Table 3:** Multiple regression analysis of factors associated with pediatric obstructive sleep apnea knowledge *: significant, B: unstandardized coefficient, SE: standard error, β: standardized coefficient, t: t-statistic, and OSA: obstructive sleep apnea

Variable	B	SE	β	t	P. value
(Constant)	8.115	1.394	—	5.822	< .0001*
Age	-.213	.255	-.055	-.838	.402
Sex	.406	.471	.044	.863	.389
Marital Status	-.063	.459	-.008	-.137	.891
Education	.891	.224	.200	3.978	< .0001*
Children	-.142	.395	-.025	-.359	.719
Knew child with OSA	1.422	.665	.107	2.137	.033*

**Figure 1:**
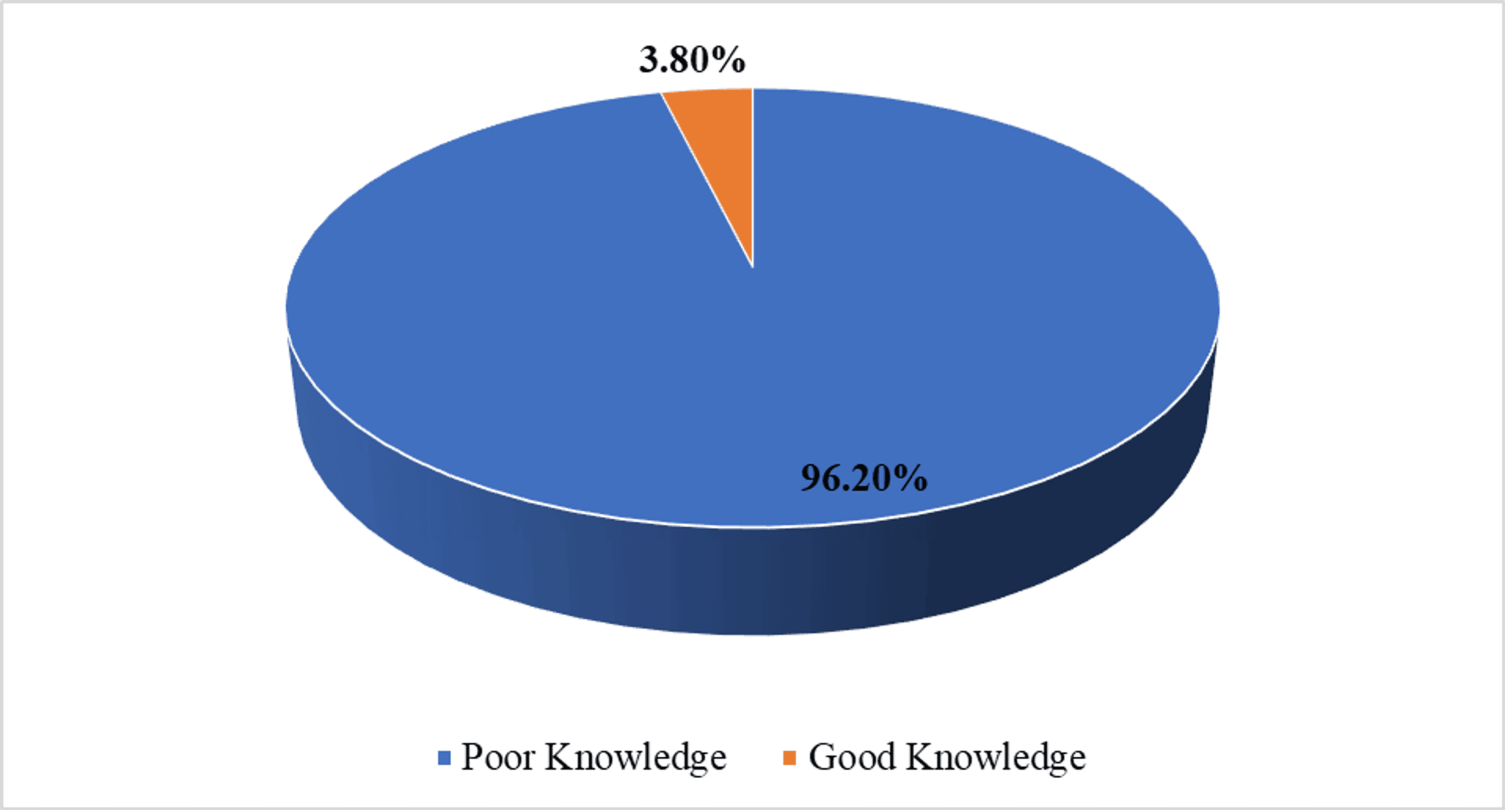
Overall knowledge score regarding OSA OSA: Obstructive sleep apnea

Figure 1 shows that a majority of the participants (378; 96.20%) had poor knowledge; only 15 (3.8%) reported good knowledge regarding OSA.

**Figure 2:**
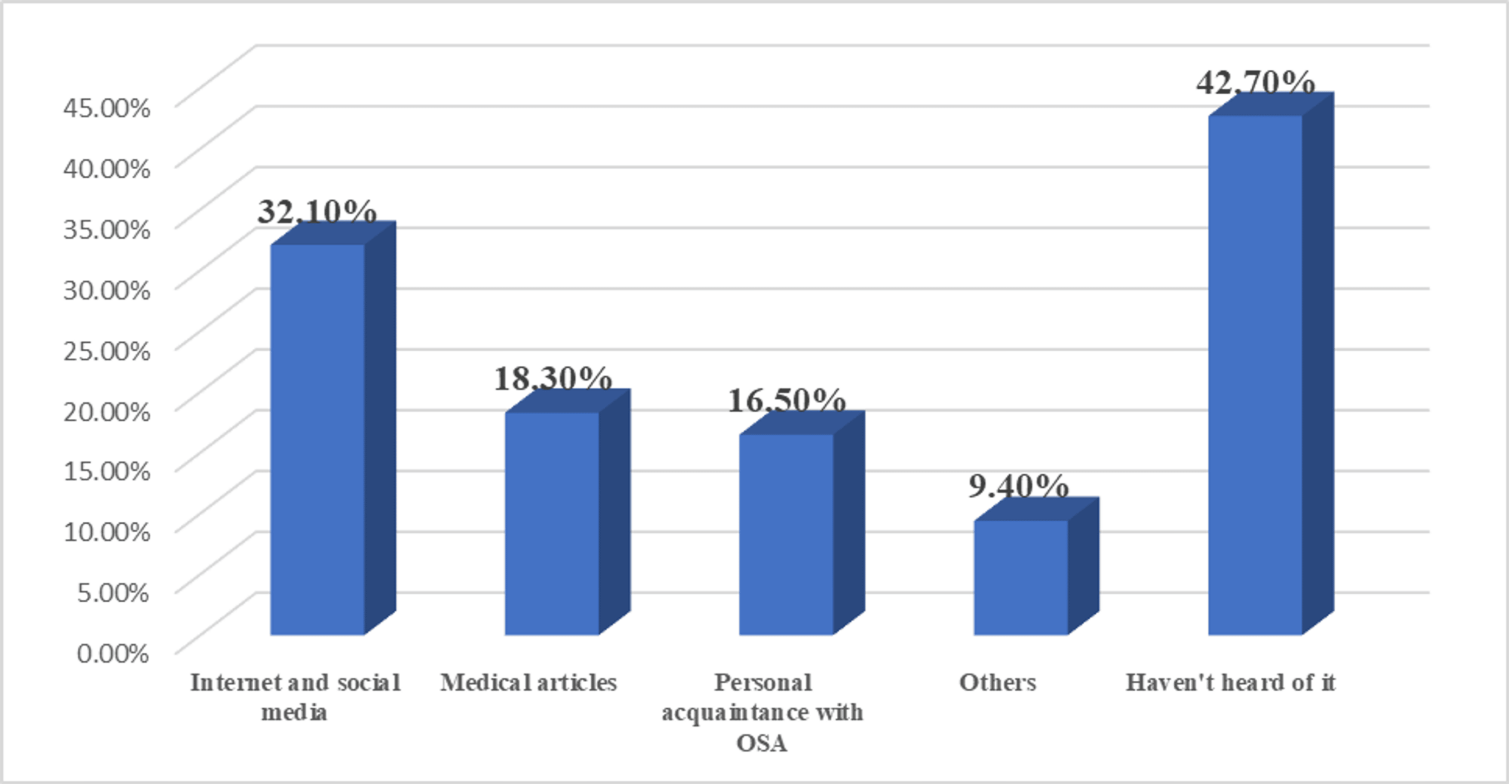
Sources of information regarding OSA OSA: Obstructive sleep apnea

According to Figure 2, the primary sources of information regarding OSA were the internet and social media (n=126, 32.1%) and personal acquaintances (n=65, 16.5%). Notably, 42.7% (n=168) of respondents indicated they had no prior awareness of the condition.

**Figure 3:**
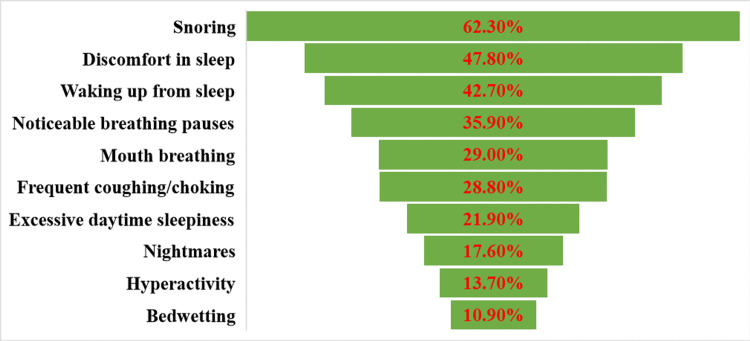
The percentage of correct answers regarding the symptoms of childhood OSA OSA: Obstructive sleep apnea

In terms of symptom recognition, Figure 3 shows that the awareness was highest for snoring (245; 62.3%), followed by discomfort during sleep (188; 47.8%) and frequent waking (168; 42.7%), while hyperactivity (54; 13.7%) and bedwetting (43; 10.9%) received the lowest recognition.

**Figure 4:**
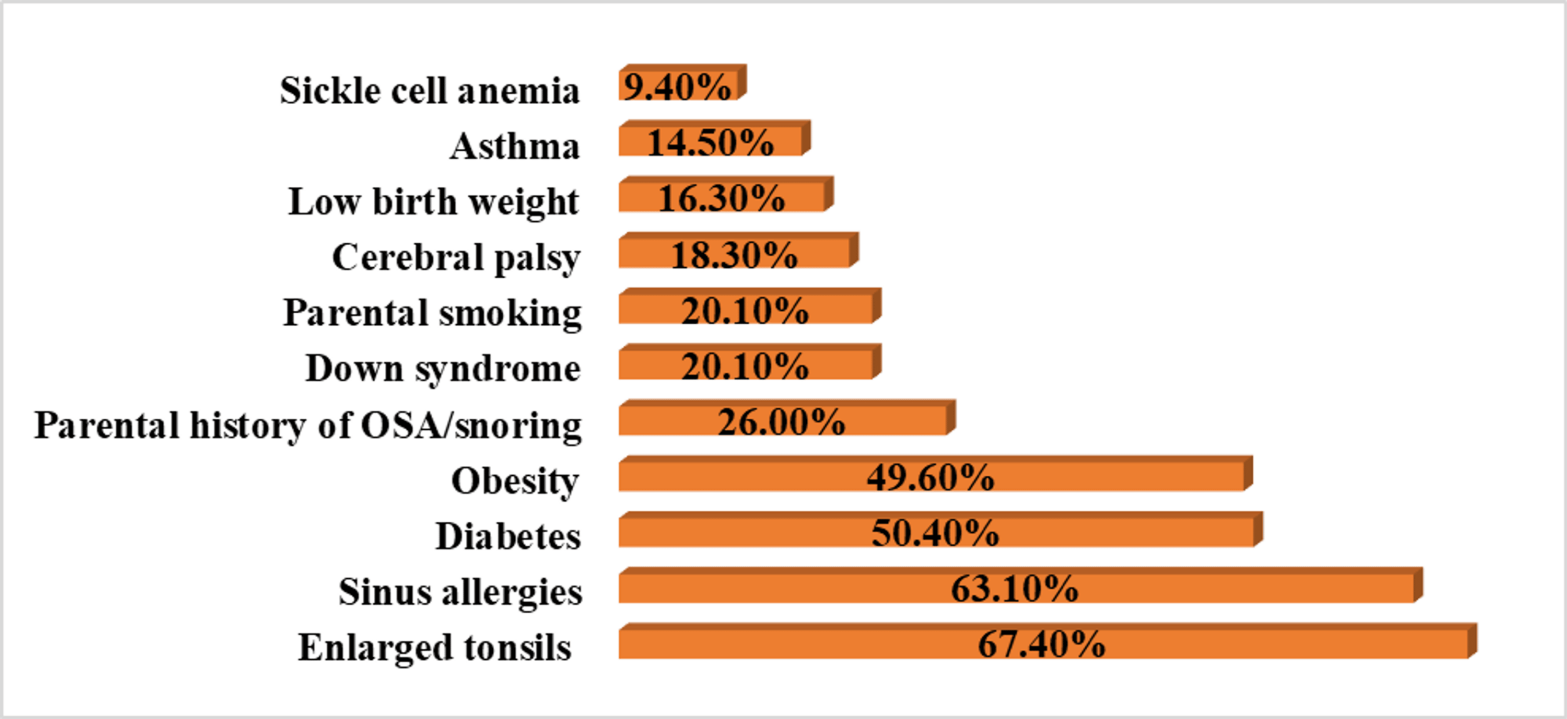
The percentage of correct answers regarding the risk factors of childhood OSA OSA: Obstructive sleep apnea

Regarding risk factors, Figure 4 shows the highest levels of awareness for enlarged tonsils and adenoids (265; 67.4%) and sinus allergies (248; 63.1%). Approximately half of the participants also recognized diabetes (198; 50.4%) and obesity (195; 49.6%). Conversely, the lowest awareness was observed for asthma (57; 14.5%) and sickle cell anemia (37; 9.4%).

## Discussion

Healthy sleep is crucial to a child's development, and OSA affects approximately 5% of children [11,12]. Our study aimed to assess the knowledge and awareness of the general population toward OSA. This study included 393 participants; 50.1% of the participants were males. This was comparable with another study in Al-Ahsa, Saudi Arabia [1] in which there was a male predominance (209; 58.1%). Over half of them are between 18 and 40 years old, and 70.7% have a bachelor's degree or higher. Similarly, research in Jazan Region [13] documented that most of the participants had a bachelor’s degree (69%).

The present study found that there were significant associations between knowledge and awareness of pediatric OSA and educational level, number of children, and marital status. This significant association between educational level and knowledge is consistent with previous studies conducted in Saudi Arabia and elsewhere. For instance, a nationwide cross-sectional survey of 16,662 participants [14] reported that education emerged as a strong predictor of OSA awareness in this study. In the Western region [15], significantly higher OSA knowledge was found among educated participants. Another cross-sectional study was done on 675 parents to assess the level of awareness about pediatric OSA among Saudi parents. It was revealed that the level of knowledge was significantly correlated to the parents’ level of education [12]. Moreover, a study in Jazan, Saudi Arabia, reported that participants with higher education degrees had higher knowledge scores toward pediatric OSA [10]. In the same line, a previous study in the central region of Saudi Arabia noted that a statistically significant association was found between marital status, education level, and OSA awareness with p-values of less than < 0.05 [16].

The present study found that there were no significant associations between knowledge and awareness of pediatric OSA and age or gender. These results agree with previous studies in Saudi Arabia [12,17], which noted that there was no significant correlation between knowledge and awareness of pediatric OSA and age. Conversely, a study in Al-Baha Region, Saudi Arabia, found that age or gender were significant associations with knowledge and awareness of pediatric OSA [18].

This study found considerable information gaps in the knowledge and awareness of the general population, with an alarming 378 (96.20%) having poor knowledge of OSA, while 15 (3.8%) have good knowledge. As a result of this finding, it appears that pediatric OSA remains poorly understood in the community, despite the increasing prevalence rates [14]. The present study's findings are consistent with previous Saudi studies in which limited awareness was reported, although it appears that the lack of knowledge in this study is even more severe than in previous studies. For example, a study in Jeddah showed that 84% of parents showed poor knowledge while 16% showed good awareness [11]. Similarly, in the Central Region of Saudi Arabia, 61.8% of participants had poor knowledge, while 38.2% had good knowledge [16]. Likewise, 66.3% of participants in southwestern Saudi Arabia had inadequate knowledge [9]. In contrast, a study from Al-Baha Region, Saudi Arabia, reported relatively better findings, with 64% of the general population classified as having adequate knowledge while 35.4% of them showed inadequate awareness [18]. Another study demonstrated that 35.1% of participants had an insufficient level of knowledge, while only 6.2% had a high level of POSA knowledge [9]. Variations in study demographics, regional context, questionnaire design, and the thresholds employed to determine adequate knowledge could all be contributing factors to these discrepancies. Internationally, the current results are also broadly in line with international studies, which also show that public knowledge of pediatric OSA is often poor. In Australia, only 30.4% of people knew that children might have OSA, which means that almost 70% of people were unaware of pediatric OSA [19]. Likewise, a study from South China found that only 47.2% of respondents were aware of childhood OSA [20]. As well as a study conducted in Lorraine, France, found that most participants had poor knowledge about OSA [21]. Another study from China was conducted, and it found that 79% of the participants had poor knowledge of OSA [22].

Regarding the main sources of information on pediatric OSA, they were the internet and social media 126 (32.1%), followed by personal acquaintance with OSA 65 (16.5%), while a considerable proportion of participants 168 (42.7%) reported that they had never heard of the condition before the survey. This pattern is in line with a number of local Saudi studies that found internet-based platforms to be the most important information source. For instance, a study conducted in Jeddah [11] reported that sources of information on OSA were the Internet and social media (36.8%). Similarly, in Jazan, a study reported that internet and social networking sites accounted for 33.33% of information sources. Likewise, in the Central Region, the internet and social media were the main source of knowledge at (31.2%) [16]. A similar trend was observed in southwest Saudi Arabia, where 49.5% of the social media users were their main sources of information on pediatric OSA [9]. These results imply that the public's main source of information regarding pediatric OSA in Saudi Arabia is the internet and social media. 42.7% of participants in this study had never heard of the condition, which also aligns with previous local studies showing substantial baseline unawareness, for example, in Jeddah, 35.9% [11]; in Jazan, 38.17% [10]; and in the Central Region, 33.9% [16]. Similar prior awareness limitations have also been documented internationally, albeit the predominant information sources may vary depending on the context. Nearly 70% of Australians were unaware of pediatric OSA, with just 30.4% of the general public aware that OSA might harm children [19]. According to the same study, family and friend communication was the most popular source of information, followed by television shows and medical specialists, rather than digital media. In South China, only 47.2% of parents had heard of childhood OSA [20].

With regard to symptom awareness recognition, it was highest for snoring, 245 (62.3%), while bedwetting, 43 (10.9%), received the lowest recognition. Similar findings were reported in Jazan [10], where snoring, discomfort during sleep, and waking from sleep were more commonly recognized than hyperactivity and nocturia. Likewise, in the Central Region, snoring was the most recognized symptom, whereas behavioral and daytime manifestations showed very low recognition [16]. Internationally, similar patterns have been observed, where awareness tends to focus on classic respiratory symptoms, whereas enuresis and behavioral problems are underrecognized [19]. Additionally, these findings align with another study by Alhawiti et al. [23], which reported that the most commonly recognized symptoms included snoring, mouth breathing, and difficulty breathing during sleep.

The present study found that most participants were aware that enlarged tonsils, adenoids, sinus allergies, diabetes, obesity, asthma, and sickle cell anemia awareness are risk factors for OSA, consistent with the findings of a previous study conducted in Jeddah [11] indicating that the majority of participants correctly identified adenoids, allergic sinusitis, enlarged tonsils, asthma, sickle cell anemia, and diabetes mellitus as risk factors for OSA. Likewise, previous studies [10,12] reported that most participants were aware of risk factors for OSA.

Study strengths and limitations

A primary strength of this study is the use of a validated instrument with high internal consistency (α= 0.82), ensuring reliable metrics. Additionally, the final sample (n = 393) met statistical power requirements, establishing the first baseline dataset for pediatric OSA awareness in the Al-Ahsa region. Conversely, the cross-sectional design limits the ability to infer definitive causal relationships. The use of an online social media (convenience sample) survey introduced selection bias, resulting in a highly educated sample (70.7% university graduates) that may not fully reflect the general population. Furthermore, self-reported data remain vulnerable to information bias that limits the generalizability of the findings.

## Conclusions

This study highlights a critical public health gap in Al-Ahsa, where 96.2% of the population exhibits deficient knowledge regarding pediatric OSA. Although nocturnal symptoms like snoring are widely recognized, over 40% of participants are entirely unfamiliar with the disorder, and significant deficits remain concerning its severe neurobehavioral, emotional, and academic complications. Furthermore, low awareness is heavily correlated with lower educational attainment, unmarried status, and larger family sizes. To mitigate these gaps, public health authorities should implement targeted digital and specialist-led campaigns, integrate brief OSA screenings into routine pediatric clinical visits, and train educators to identify daytime cognitive symptoms. Finally, conducting multi-center, stratified research is an essential step to addressing awareness variations across the Kingdom.
